# Knockdown of SET Domain, Bifurcated 1 suppresses head and neck cancer cell viability and wound-healing ability in vitro

**DOI:** 10.3906/biy-1903-71

**Published:** 2019-10-14

**Authors:** Sibel ÖZDAŞ*

**Affiliations:** 1 Department of Bioengineering, Faculty of Engineering Sciences, Adana Alpaslan Türkeş Science and Technology University, Adana, Turkey

**Keywords:** Head and neck cancer, SETDB1, qRT-PCR, western blot, siRNA, cell viability, scratch wound-healing, caspase-3 activity

## Abstract

Head and neck cancer (HNC) is the sixth most common cancer worldwide and therefore presents a global public health problem. There are no standard algorithms for the diagnosis and follow-up of the disease, and no effective current treatment approaches exist. Therefore, the discovery of new biomolecules and the design of new strategies to aid in early diagnosis is necessary, along with establishing prognostic factors of HNC. In several cancer studies, the upregulation of SET Domain, Bifurcated 1 (SETDB1) has been reported to be tumor-inducing and to indicate a cancer-invasive prognosis, leading to the modulation of genes associated with different signaling pathways; however, the literature is sparse regarding the relationship between SETDB1 and HNC. In our study, three HNC primary cell lines and their corresponding metastatic cell lines were used. The quantitative reverse transcriptase-polymerase chain reaction and western blotting data indicated that the SETDB1 mRNA and protein expression levels were higher in all metastatic cell lines compared to their primary cell lines (P < 0.05 for all). To investigate the role of SETDB1 in HNC biology, in vitro functional analyses were carried out using small interference RNA (siRNA) technology, cell viability, scratch wound-healing, and the caspase-3 activity assay of gene expression of SETDB1 to compare primary and metastatic cell lines of HNC. Metastatic cells were more susceptible to this suppression, which decreased the vitality of cells and their ability of wound-healing and induced level of caspase-3 activity (P < 0.05 for all). This functional study has shown that SETDB1 plays an important role in head and neck carcinogenesis. Therefore, SETDB1 may be an attractive therapeutic target molecule and also a potential diagnostic and prognostic biomarker in HNC.

## 1. Introduction

Head and neck cancer (HNC) is one of the most prevalent and deadly cancers in the world and therefore is an important health problem. It is composed of cancer-related structures in the head and neck, including the lips, mouth, nasal cavity, paranasal sinuses, salivary glands, oropharynx, nasopharynx, hypopharynx, and larynx (Jemal et al., 2007). The mortality rate is 0.53, and death is three times more frequent in men (Pehlivan et al., 2008). Despite many technological advances, there has been no significant change in survival; metastasis is still the main cause of death in HNC patients (Jemal et al., 2007). In most cases, however, 51% of all HNC patients have a close metastasis, while only 10.5% had distant metastases identified. The 5-year survival rate was 51% in patients with close metastases and 28% in those with distant metastases (Jemal et al., 2007).

HNC is invasive, aggressive, and characterized by a poor prognosis. In addition, other current treatment approaches, such as radiotherapy and chemotherapy, are inadequate due to their toxic effects and drastic mechanisms of action, along with traditional surgical practices that reduce patient quality of life. In addition, the high recurrence rate of HNC and its metastatic character, which brings into question the efficacy of current therapeutic approaches, suggests that alternative treatment methods are needed. Therefore, the discovery of biomaterials that can be used for the early diagnosis and follow-up of HNC and the design of new therapeutic strategies that explain potential molecular mechanisms are very important.

As with most cancers, various genetic factors play a role in the pathology of HNC (Renan et al., 1993). In addition to genetic changes, epigenetic dysregulation, including downregulation of tumor suppressor genes, activation of oncogenes and growth factors, and/or expression changes in target genes in signal pathways, play a key role in HNC’s pathogenesis (Castilho et al., 2017). However, some recent studies have shown that dysregulation of DNA methylation via aberrant *SET Domain, Bifurcated 1* (*SETDB1*) gene expression can silence critical tumor suppressor genes and induce a more aggressive tumor phenotype in HNC (Bakhtiar et al., 2015; Mochizuki et al., 2018).

Epigenetic regulation is a critical process that regulates noncoding RNA in eukaryotic cells, DNA methylation, covalent-histone modifications, and gene expression involving noncovalent mechanisms (Dawson et al., 2012). Epigenetic regulation mechanisms control the genome function together by changing structural dynamics in some regions of the chromatin and various processes such as aging, embryological development, and carcinogenesis (Sun et al., 2014). The alteration of epigenetic mechanisms including DNA methylation and histone modification is reportedly linked to cancer pathogenesis and has begun to attract the attention of therapeutic researchers in the development of targeted molecular therapeutic strategies (Dawson et al., 2012). Moreover, as an important histone modification, the histone methyltransferases (HMTs), which belong to the SET-domain protein family, have been demonstrated to be dysregulated in metabolic pathways and promote the emergence of a tumorigenic phenotype (Baylin et al., 2006). SETDB1 (SET Domain, Bifurcated 1, ESET, KMTLE) as an HMT adds three methyl groups to the lysine residue (H3K9me3) and silences the expression of important genes due to the formation of heterochromatin (Dodge et al., 2004). The human SETDB1 (Q9UPS6) is a 143.1-kDa protein encoded by the localized *SETDB1* gene on chromosome 1q21. SETDB1 is essential for embryogenesis (Matsui et al., 2010), the development (Matsui et al., 2016) and inactivation of the X chromosome, and cellular differentiation (Minkovsky et al., 2014). The overexpression of *SETDB1 *has been reported to induce the modulation of genes associated with various signaling pathways, including those for tumorigenesis and those that promote cancer invasion and progression (Ceol et al., 2011; Wong et al., 2016; Huang et al., 2018). The increased expression of *SETDB1* is correlated with HNC progression in The Cancer Genome Atlas (TCGA) (https://www.cancer.gov). However, the role of *SETDB1* in HNC biology has not yet been clarified. 

Therefore, in our study, *SETDB1* gene expression in HNC cell lines was studied at the mRNA and protein levels. In addition, we investigated the effect of its suppression on the viability, wound-healing capacity, and level of caspase-3 activity of HNC cells by *SETDB1* knockdown with small interference RNA (siRNA) technology. 

## 2. Materials and methods 

### 2.1. Cell culture

Three pairs of primary and metastatic cancer cell lines were used, and their clinicopathological characteristics are summarized in Table 1. The cell lines were seeded on Dulbecco’s modified Eagle’s medium (DMEM) (Sigma-Aldrich, Germany) along with 10% fetal bovine serum, 1% penicillin-streptomycin, 1% L-glutamine, and 0.01% Plasmocin. They were cultured in a humidified incubator with 95% air and 5% CO_2_ at 37 °C. The movement of cells and the tracing process were observed using an inverted microscope (Leica, Germany).

**Table 1:** The characteristics of the HNC cell lines.

Cell lines		Origin	Sex/age	Classification
Primary cell lines (A series)	16A	Tongue	F/77	T3N0M0/III
	42A	Laryngeal	M/43	T4N3bM0
	74A	Tongue	M/51	T3N1M0
Metastatic cell lines (B series)	16B	Neck	F/77	T3N0M0/III
	42B	Neck	M/43	T4N3bM0
	74B	Neck	M/51	T3N1M0

HNC = Head and neck cancer; M = male; F = female; TNM = tumor stage involvement size, lymph node status, distance of metastases.

### 2.2. Quantitative reverse transcription polymerase chain reaction (qRT-PCR)

Quantitative reverse transcription polymerase chain reaction (qRT-PCR) was used to detect the level of *SETDB1* gene expression in the cell lines. A High Pure RNA Isolation Kit (Roche Diagnostics, USA) was used to isolate the RNA. For the qRT-PCR, a Transcriptor High Fidelity cDNA Synthesis Kit (Roche Applied Science, Germany) was used to synthesize complementary DNA (cDNA) in a thermal cycler. Briefly, 2 µL of cDNA was mixed with 18 µL from the SYBR Green qPCR reaction kit (Roche Applied Science, Germany) for the qRT‐PCR using primer pairs (Table 2). Glyceraldehyde-3-phosphate dehydrogenase (*GAPDH*) gene expression was used for normalization of the *SETDB1* expression in qRT-PCR using the comparative C_T_ method (ΔΔC_T_) (Livak and Schmittgen, 2001). qRT-PCR was carried as described in the manufacturer’s protocol (Rotor-Gene Q 5plex HRM Platform; QIAGEN, Germany) (Sun et al., 2014).

**Table 2:** The primer sets.

Target gene	Direction	Primers
SETDB1	F	5’ TTAACACAGGCCCTGAATTTCT 3’
	R	5’ TACCCCTGTGGGTAGACACTCT 3’
GAPDH	F	5’ GAAGGTGAAGGTCGGAGTC 3’
	R	5’ GAAGATGGTGATGGGATTTC 3’

SETDB1= SET Domain, Bifurcated 1; GAPDH = glyceraldehyde-3- phosphate dehydrogenase; Forward = F; Reverse = R.

### 2.3. Western blotting

The SETDB1 protein expression level was assessed by western blotting. The confluent siRNA *SETDB*1 and control cells were washed with 1 mL of Dulbecco’s phosphate-buffered saline (DPBS) at 4 °C three times and lysed in a radioimmunoprecipitation assay (Thermo Scientific, USA). Mammalian Protein Extraction Reagent (M-PER; Thermo Scientific, USA) was used to isolate the total proteins. The concentrations of these isolated proteins were measured using the Bradford method. For western blotting, 20 µg of each protein was used. The proteins were loaded onto SDS-PAGE gel and run in ProSieve EX running buffer (Lonza Bioscience, USA) by electrophoresis. Next, the protein was transferred to membranes (Lonza Bioscience, USA). The membranes along with the transferred proteins were blocked in 5% nonfat milk and incubated with primary rabbit monoclonal antibody-SETDB1 diluted to 1:1000 (Cell Signaling, USA) and subsequently with secondary anti-rabbit IgG HRP-linked antibody. **β-Actin** was used for normalization. Protein bands were observed with ECL solution (Bio-Rad, USA) and their relative densities were assessed with densitometric analysis by using Image Lab software (Bio-Rad, USA) (Sun et al., 2011; Taylor et al., 2013; Özdaş, 2018).

### 2.4. Transient transfection 

The UT-SCC 16A and 16B and UTSCC 74A and 74B cells were seeded in media without antibiotics (1.2 × 10^5^cells/well) and treated with siRNA *SETDB1* using a transfection reagent (DharmaFECT-1, GE Healthcare, USA). The efficiency of the transient transfection in cells treated with siRNA *SETDB1* was assessed by qRT-PCR and western blotting. The manufacturer’s protocol was followed. After 24 h, the cells were harvested for further analyses. 

For transient transfection by siRNA knockdown, siRNApool technology was used, and all of the siRNAs were synthesized by Dharmacon (GE Healthcare, USA). For specific siRNAs *SETDB1*, for a nonsilencing control and *GAPDH* control, the ON-TARGETplus Human *SETDB1* siRNA-SMARTpool and Human Non-Targeting-Control Pool and Human *GAPDH*-Control Pool (GE Healthcare, USA) were respectively used (Özdaş, 2018).

### 2.5. Cell viability assay

The MTT assay (3-(4,5-dimethylthiazol-2-thiazolyl)-2,5-diphenyl-2H-tetrazolium bromide) (Sigma-Aldrich, Germany) was used to evaluate the effect of *SETDB1* on cell viability (Na et al., 2016). MTT was dissolved in DPBS (GE Healthcare, USA). For the MTT assay, after transfection for 24 h, siRNA *SETDB1* and the control cells were cultured with 100 µL of media in 96‐well plates (1–1.2 × 10^4^ cells/well) under standard conditions. The culture media were removed following incubation for 24 h, and the cells were washed with DPBS. Next, the MTT solution was added to the plate, which was kept for 4 h at 37 °C under standard incubation conditions. After incubation, the MTT solution was removed, and the formazan crystals on the plate were dissolved in 100 µL of dimethyl sulfoxide (Sigma-Aldrich, Germany). The absorbance at 570 nm was measured using a spectrophotometer (Shimadzu, UVmini-1240). 

### 2.6. Caspase-3 activity assay

To evaluate *SETDB1*’s effect on caspase-3 activity, a caspase-3 activity assay was carried out using a kit (Sigma-Aldrich, Germany) following the manufacturer’s protocol. After transfection for 48 h, the siRNA *SETDB1* and control cells were cultured into 96-well plates (1–1.2 × 10^4^ cells/well) under standard incubation conditions. The caspase activity was measured at absorbance of 405 nm using a spectrophotometer (Ko et al., 2014).

### 2.7. Scratch wound-healing assay

To evaluate *SETDB1*’s effect on wound closure, a scratch assay was performed. The siRNA *SETDB1* and control cells were cultured into six‐well plates (1–1.2 × 10^5^ cells/well). When the monolayer of cells reached confluence, scratch wounds were created with a 200-µL micropipette tip and washed with DPBS (GE Healthcare, USA). The cells were maintained under standard conditions. The wound area was documented using a Leica inverted microscope (Frankfurt, Germany) under 10× magnification at 0 h, 24 h, and 48 h, respectively. The scratch-wound area was measured using ImageJ software (Wu et al., 2016). The fractional closure of the wound area was calculated as the ratio between the relative residual wound area at the beginning and at the end of the assay. 

### 2.8. Statistical analysis

SPSS 21.0 (IBM Corp., USA) was used for all statistical analyses, and data are shown as the mean ± standard error of mean (SEM). The qRT-PCR and caspase-3 activity assay data were analyzed by Mann–Whitney U test. Student’s t-test was performed to analyze the other data. P < 0.05 was considered statistically significant.

## 3. Results

### 3.1. Overexpression of the SETDB1 gene and the SETDB1 protein in HNC cells 

**Figure 1:**
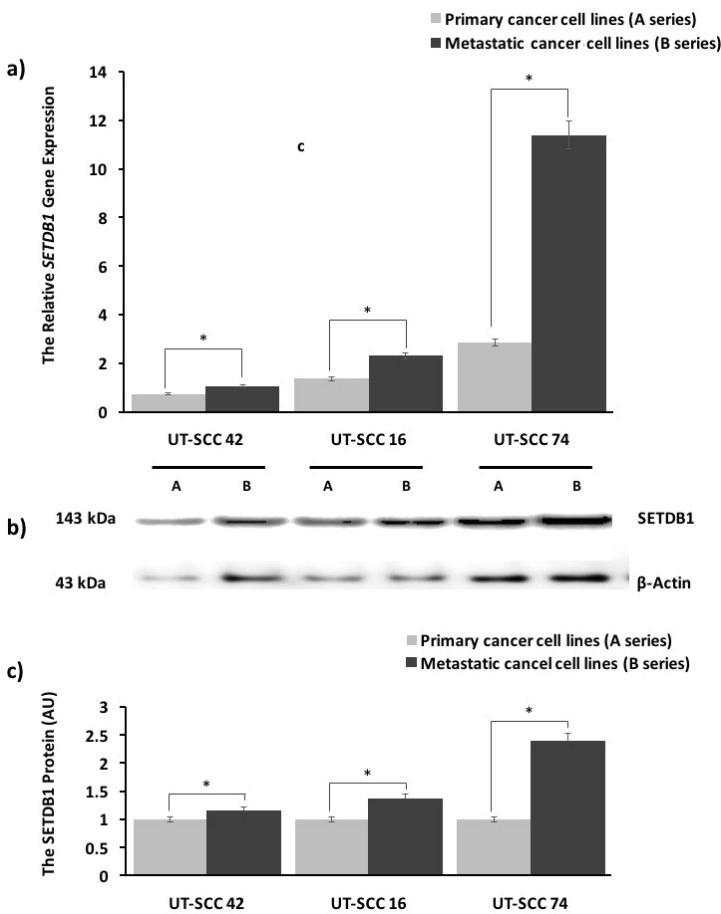
SETDB1 expression in HNC cell lines. (a) SETDB1 gene expression was evaluated using qRT-PCR at the mRNA level and with GAPDH as a housekeeping gene. The relative SETDB1 gene expression increased significantly in the metastatic HNC cell lines (UT-SCC 42B, 16B, 74B) compared to primary defects (UT-SCC 42A, 16A, 74A). GAPDH was used for normalization of qRT-PCR data. The relative fold change analysis was conducted using the ΔΔCT method. qRT-PCR data expressed the relative fold change and represent the mean ± SEM for three independent biological replicates. (b) SETDB1 protein expression was evaluated by western blotting. β-Actin was used for normalization of western blotting data. The expression of the SETDB1 protein was higher in metastatic HNC cell lines (UTSCC 42B, 16B, 74B) compared to primary cell lines (UT-SCC 42A, 16A, 74A). (c) The relative intensities of SETDB1 protein bands were evaluated densitometrically. Densitometric results showed that there was an overall statistical increase in the amount of SETDB1 protein for metastatic HNC cell lines compared to primary cell lines. Densitometric analysis was conducted using software. Densitometric analysis data were expressed as AU and as n-fold over their primary counterparts and represent the mean ± SEM for two independent biological replicates. *P < 0.05, values were compared with their primary cell lines. UT-SCC = Head and neck squamous cell carcinoma; SETDB1 = SET Domain, Bifurcated 1; GAPDH = glyceraldehyde-3-phosphate dehydrogenase; ΔΔCT = comparative CT; standard error of the mean = SEM; AU = arbitrary units.

*SETDB1 *expression was evaluated in primary and metastatic cell line pairs of HNC by qRT-PCR and western blotting because dysregulation of *SETDB1* is critical for different signaling pathways and has been suspected to stimulate cancer phenotypes (Richter et al., 2009; Sun et al., 2014; Sun et al., 2015). *SETDB1* expression was found to be significantly higher in all metastatic HNC cells than in their primary counterparts, and its highest expression level was detected in UT-SCC 74B cells by qRT-PCR (P < 0.05 for all). In metastatic cell lines, the expression of *SETDB1* compared to the primary cell lines was significantly increased by ~1.44-fold in UT-SCC 42B/42A, ~1.70-fold in UT-SCC 16B/16A, and ~3.98-fold in UT-SCC 74B/74A (respectively, P = 0.019, P = 0.004, and P < 0.001) (Figure 1a). 

The expression of the SETDB1 protein was also assessed by western blotting in HNC cells and was significantly higher in all metastatic HNC cells than in their primary counterparts. The strongest expression level was demonstrated in UT-SCC 74B cells by western blotting, similar to the qRT-PCR data (Figure 1b). According to densitometric analysis, the significant increase in the SETDB1 protein was ~1.15-fold in UT-SCC 42B/42A, ~1.36-fold in UT-SCC 16B/16A, and ~2.39-fold in UT-SCC 74B/74A (respectively, P = 0.04, P = 0.03, and P = 0.002) (Figure 1c).

## 3.2. The efficiency of knockdown of SETDB1 by siRNA in HNC cell lines 

The expression of *SETDB1* in HNC cell lines was suppressed to investigate its functional role in cellular processes using siRNA technology. The primary and metastatic HNC cell line pairs of UT-SCC 16 and UT-SCC 74 were treated with* SETDB1* siRNA or nontargeting siRNA as the control. The efficiency of the knockdown of *SETDB1* was studied with qRT-PCR within 48 h and western blotting within 72 h. 

**Figure 2:**
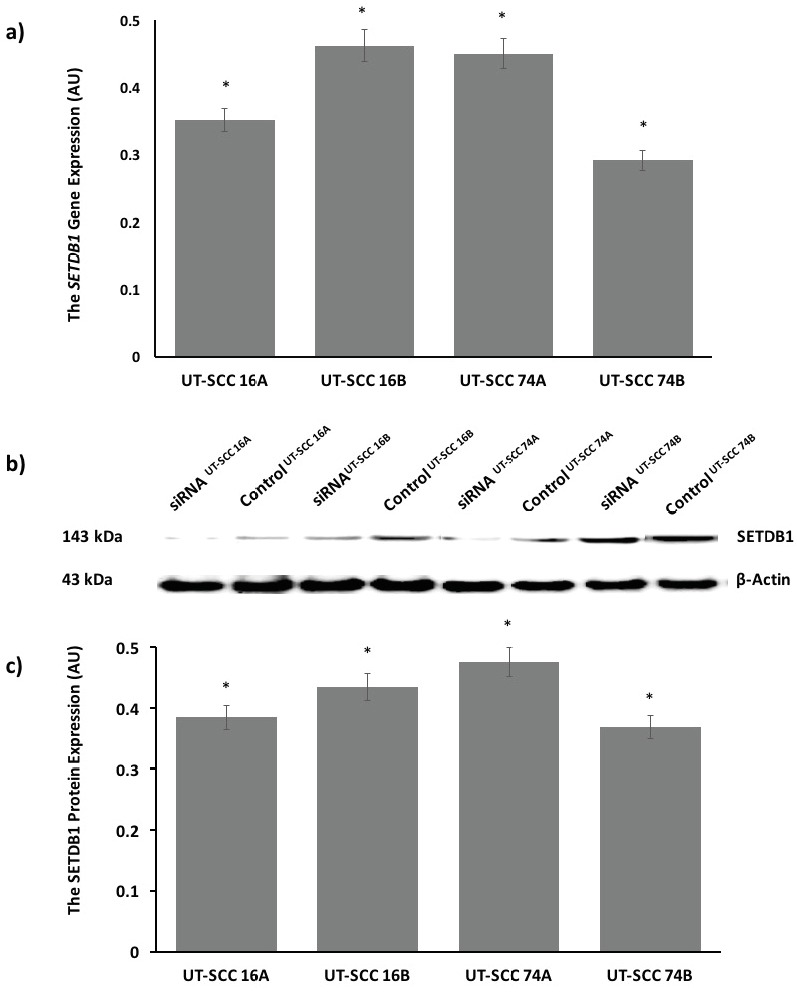
The efficiency of SETDB1 knockdown by siRNA in HNC cell lines. Control siRNA and SETDB1 siRNA were treated in UTSCC 16A, 16B, 74A, and 74B cells. (a) The transfection efficiency was analyzed after 48 h of RT-qPCR. The data indicated that the expression of the SETDB1 gene was successfully suppressed at the mRNA level in the primary and metastatic HNC cells treated with SETDB1 siRNA compared to the control siRNA. GAPDH was used for normalization of qRT-PCR data. The relative fold change analysis was conducted by using the ΔΔCT method. qRT-PCR data were expressed as AU and as n-fold over their control-siRNA and represent the mean ± SEM for three independent biological replicates. (b) Western blotting analysis showed that the expression of the SETDB1 gene was suppressed at protein level in both the primary and metastatic HNC cells treated with SETDB1 siRNA compared to the control siRNA. β-Actin was used for normalization of western blotting data. siRNAUT-SCC and ControlUT-SCC indicate the protein levels of SETDB1 in SETDB1 siRNA and control siRNA cell lines, respectively. (c) The relative intensities of SETDB1 protein bands were analyzed densitometrically. Densitometric results showed that there was an overall statistical decrease in the amount of SETDB1 protein in HNC treated with SETDB1 siRNA compared to the control group of siRNA. Densitometric analysis was conducted using software. Data of the densitometric analysis were expressed as AU and as n-fold over control-siRNA and represent the mean ± SEM for two independent biological replicates. *P < 0.001, values were compared with their control group of siRNA. HNC = Head and neck cancer; UT-SCC = head and neck squamous cell carcinoma; SETDB1 = SET Domain, Bifurcated 1; siRNA = small interference RNA; GAPDH = glyceraldehyde-3-phosphate dehydrogenase; ΔΔCT = comparative CT; standard error of the mean = SEM; AU = arbitrary units.

The relative expression of* SETDB1* in HNC cell lines transfected with *SETDB1* siRNA compared to control siRNA**by qRT-PCR was found to be significantly decreased (P < 0.001 for all) (Figure 2a). Moreover, the expression of the SETDB1 protein in all HNC cell lines treated with *SETDB1* siRNA was significantly repressed compared to the control group of siRNA**as obtained through**western blotting and its densitometric analysis (P < 0.001 for all) (Figures 2b and 2c).

## 3.3. Knockdown of SETDB1 by siRNA decreased cell viability but induced activation of caspase-3 in HNC cells 

**Figure 3:**
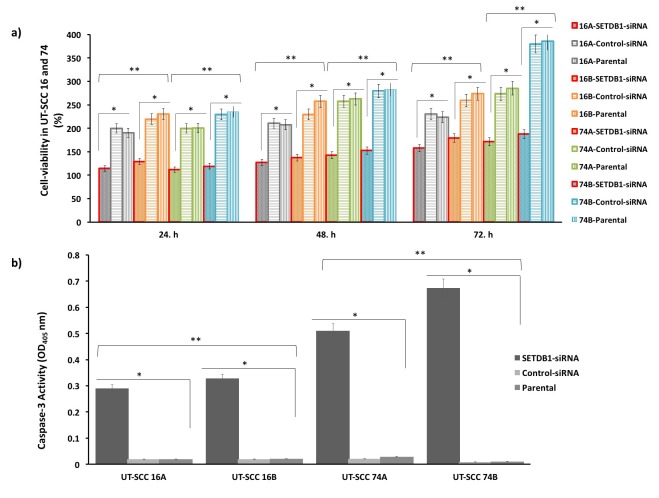
The effect of SETDB1 knockdown by siRNA on cell viability and apoptotic status in HNC cells. (a) The relative cell viability was evaluated by MTT up to 72 h. Cell viability in primary UT-SCC 16A and 74A, and metastatic UT-SCC 16B and 74B HNC cell lines by siRNA compared to the control siRNA and parental cell lines was significantly reduced. The metastatic HNC cell lines were more sensitive to SETDB1 knockdown than the primary cell lines. MTT data are presented as percentage of viable cells and represent the mean ± SEM for three independent biological replicates. (b) The apoptotic status in HNC cells by caspase-3 activity assay. Apoptotic activity was induced in the primary UT-SCC 16A and 74A and metastatic UT-SCC 16B and 74B HNC cells treated with SETDB1 siRNA compared to the control siRNA and parental cell lines. Caspase-3 activity was calculated as OD and represents the mean ± SEM for three independent biological replicates *P < 0.001, values were compared with their control siRNA and parental cell lines. **P < 0.05, values were compared with their primary cell lines. HNC = Head and neck cancer; UT-SCC = head and neck squamous cell carcinoma; SETDB1 = SET Domain, Bifurcated 1; siRNA = small interference RNA; standard error of the mean = SEM; OD = optical density.

To evaluate the effect of *SETDB1 *knockdown on cell vitality, the MTT assay was carried out using HNC primary (UT-SCC 16A, 74A) and metastatic (UT-SCC 16B, 74B) cell lines treated with *SETDB1* siRNA, control siRNA, and parent cells after knockdown at 48 h. The HNC cell vitality was measured at 0 h, 24 h, 48 h, and 72 h. The cell viability of the control siRNA and the parent cells in all cell lines at each measurement time was similar; no differences were observed between the groups (P > 0.05). However, the cell viability in HNC primary (UT-SCC 16A, 74A) and metastatic (UT-SCC 16B, 74B) cell lines treated with* SETDB1* siRNA in comparison with their control siRNA and parent cell counterparts was remarkably reduced in all measurements (P < 0.001 for all). In addition, the cell viability of HNC metastatic (UT-SCC 16B, 74B) cells compared with primary (UT-SCC 16A, 74A) cells in all cell lines treated with *SETDB1* siRNA was lower at 24, 48, and 72 h (P < 0.05 for all) (Figure 3a).

A caspase-3-activity assay was performed to assess the effect of *SETDB1* knockdown on the activation of caspase-3 in HNC primary (UT-SCC 16A, 74A) and metastatic (UT-SCC 16B, 74B) cell lines treated with *SETDB1* siRNA, control siRNA, and parent cells. The caspase-3 activity in the control siRNA and parent cells in all cell lines was not different in the groups (P > 0.05). The levels of caspase-3 activity were significantly induced in HNC cell lines transfected with *SETDB1* siRNA compared to control siRNA and parent cells**by ~0.29-fold for primary UT-SCC 16A and by ~0.32-fold for UT-SCC 74A, and by ~0.51-fold for metastatic UT-SCC 16B and ~0.67-fold for UT-SCC 74B (for all P < 0.001). Moreover, the activation of caspase-3 rate in HNC metastatic cells (UT-SCC 16B, 74B) compared with primary cells (UT-SCC 16A, 74A) in all cell lines treated with *SETDB1* siRNA was enhanced (P < 0.05 for all) (Figure 3b).

## 3.4. Knockdown of SETDB1 by siRNA decreased rate of wound closure in HNC cells

**Figure 4:**
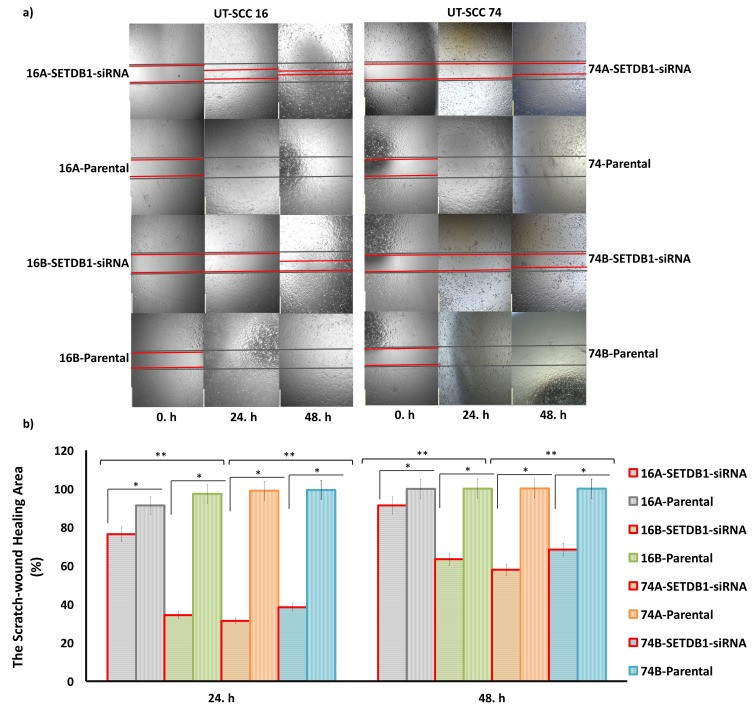
The efficiency of SETDB1 knockdown on wound closure in HNC cells. (a) The cell wound-healing abilities of SETDB1 siRNA and parental cell lines were evaluated by using scratch wound-healing assay for 48 h. The scratch wound-healing assay demonstrated that the cell wound-healing ability was decreased in primary UT-SCC 16A and 74A, and metastatic UT-SCC 16B and 74B HNC with treated SETDB1 siRNA compared to parental cell lines (10× magnification) (scale bar: 500 μm). The green and red lines represent the edges of the wound at the beginning and at the end of the assay, respectively. (b) The rate of wound closure in HNC cell lines transfected with SETDB1 siRNA compared to their parental cells at 24 h and 48 h was found to be significantly decreased. The scratch-wound area was measured using software. The relative scratch-wound area was calculated as the ratio of the wound area’s relative closure at the beginning and at the end of the assay. The scratch wound-healing assay data are presented as the percentage of the fractional closure of the wound area and represent the mean ± SEM for three independent biological replicates. *P < 0.001, values were compared with their parental cell lines. **P < 0.05, values were compared with their primary cell lines. HNC = Head and neck cancer; UT-SCC = head and neck squamous cell carcinoma; SETDB1 = SET Domain, Bifurcated 1; siRNA = small interference RNA; standard error of the mean = SEM.

A scratch wound-healing assay was performed 48 h after transfection to investigate the effect of *SETDB1 *knockdown on wound closure in HNC primary (UT-SCC 16A, 74A) and metastatic (UT-SCC 16B, 74B) cell lines treated with *SETDB1* siRNA, control siRNA, parent cells, and the migrated cells were imaged at 0 h, 24 h, and 48 h. The rate of wound closure in HNC primary (UT-SCC 16A, 74A) and metastatic (UT-SCC 16B, 74B) cells treated with* SETDB1* siRNA in comparison with their parental cell lines was remarkably decreased at 24 h and 48 h (P < 0.001 for all). In addition, the rate of wound closure in HNC metastatic cells (UT-SCC 16B, 74B) compared with primary cells (UT-SCC 16A, 74A) in all cell lines treated with *SETDB1* siRNA was reduced (P < 0.05 for all) (Figure 4a). 

The rate of wound closure in HNC cell lines transfected with *SETDB1* siRNA compared to their parental cells at 24 h and 48 h was found to be significantly decreased by ~0.83-fold and ~0.91-fold for primary UT-SCC 16A, ~0.31-fold and ~0.57-fold for UT-SCC 74A, ~0.35-fold and ~0.63-fold for metastatic UT-SCC 16B, and ~0.38-fold and ~0.68-fold in UT-SCC 74B (P < 0.05 for all) (Figure 4b).

## 4. Discussion

HNC is one of the most common deadly cancers and therefore presents a growing global public health problem. HNC’s molecular mechanism has not yet been explained because no diagnostic follow-up standard algorithms are available. Alternative strategies for investigating the molecular basis of HNC’s pathogenesis are needed because current treatment approaches are insufficient and lead to low quality of life, high toxicity, and the development of drug resistance. Therefore, the discovery of prognostic-diagnostic biomarkers and potential therapeutic and molecular targets is very urgent (Jemal et al., 2007).

Epigenetic changes, such as excessive histone modifications, have recently been linked to cancer invasion and progression (Zeng et al., 2010). HMT family members, which were first defined in 2002, allow euchromatic genes to mutate by mediating histone modification and complexing with proteins, such as SETDB1, CAP-1, HP1, DNMT3A/B, MBD1, and HDAC1/2 (Fritsch et al., 2010). SETDB1 proteins are associated with many biological processes, such as Barr bodies and embryogenesis, as well as gene silence (Matsui et al., 2010; Minkovsky et al., 2014; Song et al., 2015). Recent reports have revealed the SETDB1 protein’s functional role in human carcinogenesis (Sun et al., 2015). Today, SETDB1 is an important research area because its functional role allows it to act as a therapeutic molecule in cancer treatment (Karanth et al., 2017). Although some nonspecific inhibitors block the function of the SETDB1 protein, there is no specific agent effective yet, so the study of the role of SETDB1 and its molecular interactions in various cancers can help improve the therapeutic targeting of SETDB1 in the clinic (Rodriguez-Paredes et al., 2014). In the current study, to investigate the function of *SETDB1* in HNC biology, the gene expression in cell lines was suppressed and we analyzed the vitality, wound-healing ability, and level of caspase-3 activity of cells.

In our study, we determined *SETDB1* gene and protein expression in HNC in vitro. The level of mRNA and protein expression of the *SETDB1* gene in the HNC cell lines was higher in each metastatic line compared to its primary cell line. Studies have reported that the expression of *SETDB1* significantly increases the risk of melanoma (Ceol et al., 2011), lung cancer (Lindgren et al., 2012), urothelial carcinoma (Wong et al., 2012), glioma (Spyropoulou et al., 2014), ovarian cancer (Hua et al., 2014), prostate cancer (Sun et al., 2014), breast cancer (Liu et al., 2015), hepatocellular carcinoma (Wong et al., 2016), colorectal cancer (Chen et al., 2017), and solid tumors (Huang et al., 2018). With the increased activity of *SETDB1*, the excess trimethylation of various tumor suppressors may block gene expression, inducing cancer invasion and metastasis. TCGA data from 44 nontumorous tissues and 522 HNC tissues were analyzed. The *SETDB1* gene expression status was significantly higher for HNC samples (log2PCM = 5.094) than normal samples (log2PCM = 4.974) (P = 0.0086) (https://www.cancer.gov). Kaplan–Meier analysis also revealed that the increased expression of *SETDB1* was significantly correlated with the 5-year survival rate (50%) of patients with HNSCC (log-rank test, P = 0.009) (https://www.proteinatlas.org). Considering this evidence, we believe that *SETDB1* may be an attractive biomarker that demonstrates the prognosis of the disease by acting as an oncogene of the solid tumor HNC cell lines.

In this study, *SETDB1*’s upregulation of mRNA and protein was monitored in HNC cells. To understand the functional relationship of *SETDB1 *with HNC and to determine its potential as a therapeutic target, *SETDB1 *gene expression was suppressed using siRNA technology. The gene expression level of *SETDB1* was reduced in knockdown cancer cells. It has been observed that the level of caspase-3 activity, cell viability, and cell wound-healing ability change in HNC cells after successful transfection activity.

Sun et al. reported that *SETDB1 *induces proliferation and migration of lung carcinogenesis cells by arranging WNT signaling (Sun et al., 2015). Overexpression of *SETDB1* was detected in prostate cancer in clinical and preclinical studies, and the cancer phenotype was suppressed by siRNA in *SETDB1 *knockdown cells (Sun et al., 2014). In colon, lung, and liver cancers, *SETDB1* methylated and silenced T53 in the promoter region, but apoptosis was stimulated in* SETDB1* knockdown cells (Sun et al., 2015; Wong et al., 2016).

 In addition, gliomas (Spyropoulou et al., 2014), hepatocellular carcinoma (Wong et al., 2016), nasopharyngeal carcinoma (Huang et al., 2018), solid tumors (Richter et al., 2009), and lung cancer (Rodriguez-Paredes et al., 2014) have been observed to decrease the viability and wound-healing ability of *SETDB1* knockdown HNC cells in various cancer types, which is in parallel with studies reporting suppressed *SETDB1* expression with siRNA; P16-INK4A is a tumor suppressor protein that plays a role in cell cycle regulation (Wang et al., 2003). It has been reported that P16-INK4A in *SETDB1* knockdown cells with increased expression of promoter methylation decreased active cell viability again (Kostaki et al., 2014). In addition, Chiba et al. demonstrated that cell viability in a hepatocellular carcinoma control with and without *SETDB1* knockdown was similar (Chiba et al., 2015). There was no significant decrease in cell viability in carcinoma cells, but Wu et al. reported that *SETDB1* expression in metastatic lung cancer cells is low and inhibits cell migration by inhibiting the polymerization of actin (Wu et al., 2014). According to Na et al., *SETDB1* knockdown increased cell viability in lung cancer (Na et al., 2016). The effect of *SETDB1* knockdown may be different according to the type of cancer and may play a more critical role in the cancer’s progression.

The scratch wound-healing assay is a classical in vitro technique to investigate the cell motility that is known as sheet migration (Rorth, 2012). The epithelial-mesenchymal transition markers, extracellular matrix proteins, and genes that are associated with rearranging the actin cytoskeletal are important to provide cell–cell adhesion and cell–surface adhesion, and furthermore, it is closely linked with signaling pathways involving cell migration (Clause and Barker, 2013). Studies reported that SETDB1 may regulate cell motility and migration through the adhesive molecules; however, the relationship among them is not exactly clear (Wu et al., 2014; Ryu et al., 2019). In the present study, the knockdown of *SETDB1 *in HNC cells reduced the rate of wound-healing. However, *SETDB1 *knockdown in HNC cells with the underlying mechanisms of cell motility remains to be revealed.

The knockdown of *SETDB1* increased the apoptosis rate in various cancer types (Richter et al., 2009; Spyropoulou et al., 2014; Wong et al., 2016; Huang et al., 2018). Moreover, Shinoda et al. demonstrated the relationship between SETDB1 and caspase-3 (Shinoda et al., 2016). However, the role of SETDB1 in antiapoptotic mechanisms has not been completely understood yet, but it may interact with apoptotic pathways involving caspase-3 (Porter and Jänicke, 1999). In the current study, *SETDB1* knockdown led to increased levels of caspase-3 activity. Therefore, further studies are needed to assess the effect of *SETDB1* on apoptotic machinery in HNC cells.

Although there are different histological types of HNC, more than 90% of them are squamous cell carcinoma (SCC); 27% of these are laryngeal, while 24% are reported to occur in the oral cavity (Lin et al., 2007). However, most HNC cell lines are well characterized in our study, and we know many specific features, such as genetic and epigenetic marker panels, STR profiles, karyotype, radiation sensitivity, cisplatin sensitivity, status of the P53 mutation, antigen and integral expression, and activation (Van et al., 1994; Zhao et al., 2011). Although the cell line models we chose reflect the general characteristics of HNC, they each have genetic phenotypic differences. Although our study is preclinical and demonstrated the importance of the function of *SETDB1 *in HNC, it did not explore how it stimulates carcinogenesis through various pathways.

The SETDB1 protein is specifically H3K9me3 and silences the euchromatic genes (Dodge et al., 2004). Furthermore, dysregulation of H3K9me3 through excessive levels of *SETDB1* mRNA expression might silence some of the critical genes and contribute to tumorigenesis (Karanth et al., 2017). Several studies showed that *SETDB1* interacts with epigenetic modifiers such as H3K9me3, DNA methyltransferase 3, and CCCTC-binding factor in a direct/indirect way and regulates the epigenetic mechanisms. However, comprehensive insight into the expression and associations of SETDB1 with epigenetic modifiers and its effect on the epigenome in HNC is still required. For this reason, further studies applying technological methods, which include well-defined clinical samples, to potential targets of* SETDB1* are needed to clarify the molecular mechanisms underlying its functional role in HNC cells. 

Here, we conducted a preliminary study to explore the expression of *SETDB1* in HNC cell lines. We found that the overexpression of *SETDB1* in HNC metastatic lines plays a significant role in cancer development. In addition, our study showed that metastatic HNC cells are more susceptible to *SETDB1* suppression by specific siRNA, decreased cell vitality and wound-healing ability, and activation of caspase-3 induced in vitro. We suggest that targeting *SETDB1* that catalyzes histone modification in HNC treatment will be useful and may be a potentially diagnostic and prognostic biomarker. Further functional studies are required to elucidate and verify this potential.
